# Migration inhibitory factor and cluster of differentiation 74‐mediated dendritic cell apoptosis exacerbates acute acetaminophen‐induced liver injury

**DOI:** 10.1002/iid3.840

**Published:** 2023-04-17

**Authors:** Zezhou Wu, He Luan, Jinghui Huang, Boming Liao, Fang Xiao

**Affiliations:** ^1^ Department of Infectious Diseases The First Affiliated Hospital of Guangxi Medical University Nanning China

**Keywords:** acute liver injury, apoptosis, dendritic cells, macrophage migration‐inhibitory factors

## Abstract

**Introduction:**

We investigated the role of macrophage migration inhibitory factor (MIF) on dendritic cells (DC) during acetaminophen (APAP)‐induced acute liver injury (ALI) in mice.

**Methods:**

First, we randomly divided the mice into experimental (ALI model) and control groups, then intraperitoneally injected 600 mg/kg of APAP or phosphate‐buffered saline, respectively. Then, we collected liver tissue and serum samples to evaluate liver inflammation using serum alanine aminotransferase level and hematoxylin and eosin (H&E) staining of liver tissues. Flow cytometry was used to identify changes in the quantity and percentage of DCs, as well as the expression of cluster of differentiation (CD) 74 and other apoptosis‐related markers in the liver. Next, we randomly divided the mice into APAP‐vehicles, APAP‐bone marrow‐derived dendritic cells (BMDCs), APAP‐MIF, APAP‐IgG (isotype immunoglobin G antibody) groups (four mice per group), after APAP injection, we injected control extracts, BMDCs, mouse recombinant MIF antibodies, or IgG antibodies into the tail vein. Lastly, the severity of the liver injury and the number of DCs were assessed.

**Results:**

The APAP‐induced ALI mice had increased hepatic MIF expression but significantly lower amounts of hepatic DCs and apoptotic DCs than healthy mice; CD74 expression on the HDCs also increased markedly. Supplementing APAP‐induced ALI mice with BMDCs or MIF antibodies significantly increased the number of hepatic DCs compared with the control mice, alleviating liver damage.

**Conclusion:**

The MIF/CD74 signaling pathway may mediate hepatic DC apoptosis and promote liver damage.

## Graphical abstract

Migration inhibitory factor and cluster of differentiation 74‐mediated dendritic cell apoptosis exacerbates acute drug‐induced liver failure.

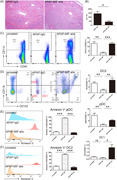

## 1 INTRODUCTION

Acute liver failure (ALF) is defined as massive and subacute‐massive hepatic tissue necrosis leading to hepatic insufficiency in the short term and is induced by several etiological agents. Drugs and their metabolites, especially excessive acetaminophen (APAP) and alcohol, and viral hepatitis are the most common causes of ALF worldwide.^1^ Abnormal immunoreactions also cause drug‐induced liver injury (DILI) in specific conditions, but these mechanisms remain unclear.^2^

Hepatic dendritic cells (HDCs) are a considerable component of immune cells in the liver; they maintain immunological tolerance and mediate immune clearance. There are three HDC subtypes: myeloid (mDC), classic, and plasmacytoid (pDCs). pDCs regulate the secretion of interferon (IFN)‐α, IFN‐β, and other cytokines. mDCs are further subdivided into DC1 and DC2 groups, promoting T helper 1 and T helper 2 cellular immunity, respectively.^3^ Specific signals in the local inner environment of the liver affect the status and function of HDCs. These changes promote liver restoration or tissue integrity after an injury. However, they can also cause gradual liver deterioration in certain diseases, such as hepatitis, fibrosis, cirrhosis, and liver cancer.^4^, ^5^, ^6^, ^7^ HDCs have a protective effect after acute liver injury (ALI),^8^ but the specific regulatory mechanisms remain unclear.

Migration inhibitory factor (MIF) is a nonassociated ligand for chemokine receptors that binds a cluster of differentiation (CD) 74 receptors (high‐affinity receptors) on the cell membrane to mediate the activation of multiple signaling pathways. MIF is a pleiotrophin with cytokines, endocrine molecules, chaperones, and enzymatic properties.^9^ Furthermore, MIF is a proinflammatory factor that mediates inflammatory liver damage in different liver diseases.^10^ The connection between MIF and HDCs in ALF has not been reported yet. However, a few studies on the relationship between MIF and HDC in other diseases (e.g., cancer, parasitic infections, and diabetes) determined that the MIF/CD74 signaling pathway regulates the functional status of DCs, but the regulation capacity varies greatly.^11^, ^12^, ^13^

This study investigated MIF and CD74 (the surface MIF ligand) expression and the HDC number and subtypes in the liver in an APAP‐induced ALI mice model to clarify the immunological pathogenesis of ALF and explore treatment possibilities.

## 2 MATERIALS AND METHODS

### 2.1 Materials

#### 2.1.1 Experimental animals

Forty specified pathogen‐free 6–8‐week‐old female C57BL/6 J mice weighing 18–20 g were purchased from and raised in the Animal Experimental Center of Guangxi Medical University. The mice were fed based on an alternating 12‐h circadian rhythm and housed in 20 ± 2°C temperatures with 50%–70% humidity.

#### 2.1.2 Drugs and reagents

The drugs and reagents were purchased from the following companies: APAP (MCE); alanine aminotransferase (ALT/GPT) detection kit (Nanjing Jicheng); protein marker (Cymefield Inc.); Sodium dodecyl‐sulfate polyacrylamide gel electrophoresis (SDS‐PAGE) Gel Rapid Configuration Kit (cat#: P0012AC; Beyotime) and RIPA cracking liquid (Beyotime); enhanced chemiluminescence substrate, rabbit anti‐MIF polyclonal antibody (cat#: abs135924; absin), and collagenase 4 (Solebo Technology Co., Ltd.); Prepoll (Shanghai Yisheng Biotechnology Co., Ltd.), erythrocyte lysate (Zhuoyi Biological Co., Ltd.); anti‐mouse CD45‐PE‐CY7 (BD), anti‐mouse Cd11c‐pe (BD), anti‐mouse‐CD74‐ApC (BD), anti‐mouse‐CD11B‐APC‐CY7 (BD), anti‐mouse‐CD103‐percP (BD), annexin‐v‐FITC/7‐AAD fluorescence double staining apoptosis detection kit (BD) (Elabscience); recombinant murine granulocyte‐macrophage colony‐stimulating factor (GM‐CSF) (cat#: 315‐03), and recombinant murine interleukin (IL‐4) (cat#: 214‐14; Peprotech); standard fetal bovine serum (FBS), penicillin‐streptomycin mixture (Servicebio); RPMI‐1640 medium (Gibco).

#### 2.1.3 Equipment

The following instruments were used: microscope (model#: BX53F+DP73+cellSens Dimension; OLYMPUS); microplate reader (model#: Thermo multiskan; Thermo Fisher Scientific); western blot analysis equipment (model#: Bio‐Rad; Hercules), Odyssey dual‐color infrared fluorescence imaging system (model#: Fluorchem M; ProteinSimple), medical ultra‐clean table (model#: SW‐CJ‐1FD; Airtech), flow cytometer (model#: FACSVerse; BD Biosciences), and cell incubator (Galaxy 170 S; New Brunswick).

### 2.2 Methods

We established an APAP‐induced ALI mouse model by overdosing the mice with 600 mg/kg of APAP. A 50 mg/mL APAP injection solution was prepared by dissolving APAP in sterile phosphate‐buffered saline (PBS). The mice were randomly divided into experimental and control groups and injected intraperitoneally with the APAP solution or PBS, respectively. Five mice from the experimental group were randomly selected 6, 12, 24, 48, and 96 h after the injection to collect liver and peripheral blood (drawn from the eyeball vein) samples; the serum was separated by centrifugation. Extracting part of the liver tissue was performed to prepare 10% tissue homogenate, then centrifuged to obtain supernatant. The same tissue portion was fixed with paraformaldehyde solution for 48 h and then stained.

Next, we randomly divided a group of the APAP‐induced ALI mice into control and MIF antibody groups. One hour after the intraperitoneal APAP injection, 50 µg of immunoglobin G antibodies (control) or 50 µg of MIF antibodies were injected into the tail vein under high pressure. Liver and peripheral blood samples were collected 11 h later.

#### 2.2.1 Serum ALT detection and pathological observations

Serum ALT levels were measured at 510 nm using a microplate reader based on the standard curve formula and the ALT/GPT detection kit instructions. The pathology of the hematoxylin and eosin (H&E)‐stained liver tissues was observed using an Olympus microscope.

#### 2.2.2 Extracting liver parenchymal mononuclear cells

Liver nonparenchymal mononuclear cells (NPCs) were extracted by hepatic portal vein perfusion digestion. First, we perfused the portal vein with a calcium‐free balanced solution followed by type IV collagenase (0.05%) at 37°C until the liver became white. Next, we digested and filtered the cell suspension with a 100 µm filter, then performed density gradient centrifugation. This method began with centrifugation at a low speed (50*g*), then the supernatant was collected. Next, 70% Percoll and 30% Percoll separation solutions were added to the supernatant, then centrifuged again. The cells of interest were collected from the space between the 70% and 30% solutions.^14^

#### 2.2.3 Flow cytometry detection of DCs in the liver

Liver NPCs were counted and resuspended in flow buffer; approximately 1 × 10^7^ cells/mL was required. After resuspension, 1 × 10^6^ cells were added to a flow tube and vortexed evenly with the surface‐labeled antibody or its isotype control (just as in the above text) in sequence following incubation at room temperature in the dark for 45 min. The samples were analyzed on the specific machine that can conduct data analysis and processing through FlowJo software version 10 (BD Life Sciences).

#### 2.2.4 MIF expression in liver tissue

Total protein was extracted from the liver tissue for western blot analyses. First, the initial protein concentration was estimated by bicinchoninic acid assay, and then the concentrations were adjusted to ensure equal protein concentrations per group. Next, the proteins were denatured, and 30 mg/mL of protein per sample were run on a 12% SDS‐PAGE. The samples were transferred and fixed to a membrane, then incubated with their respective primary antibodies (MIF: 1:1000 dilution or glyceraldehyde 3‐phosphate dehydrogenase [GAPDH]: 1:2000 dilution) overnight at 4°C. Next, the membranes were washed, then incubated with horseradish peroxidase‐labeled secondary antibodies (1:1000 dilution) for 50 min. Then, images were taken using a film scanner. The grey values of the protein bands from the images were quantified using GAPDH as an internal reference.

#### 2.2.5 Acquisition and adoptive transfer of mouse bone marrow‐derived DCs (BMDCs)

The femur and tibia of the mice were cut and irrigated at both ends to collect bone marrow cells. The red blood cells were lysed, then the bone marrow cells were cultured in RPMI‐1640 medium containing 10% FBS, 30 ng/mL of GM‐CSF, and 20 ng/mL of IL‐4. The medium containing GM‐CSF and IL‐4 was replaced with a half‐exchange solution every other day. Mature DCs were obtained 8 days later. The CD11c antibody was used for flow cytometry detection to determine the BMDC purity and GT 90%. The BMDCs were resuspended in sterile PBS at 10^7^ PCS/mL density.

Six hours after APAP injection, mice were divided randomly into two groups (four mice per group).^15^ (1) APAP + vehicle: mice were injected with 200 µL of saline solution used for BMDCs through the tail vein. And (2) APAP + DC: mice were adoptive transferred with 2 × 106 BMDCs through the tail vein. Liver tissue and peripheral blood were collected 6 h later for analyses.

#### 2.2.6 Statistical analyses

All statistical analyses were performed with GraphPad Prism version 8.0 (GraphPad Software). The independent‐sample *t* tests and single‐factor analysis of variance analysis results are expressed as means ± standard deviations. A *p* < 0.05 was considered statistically significant.

## 3 RESULTS

### 3.1 MIF expression rises along with the progression of APAP‐induced liver injury

**Figure 1:**
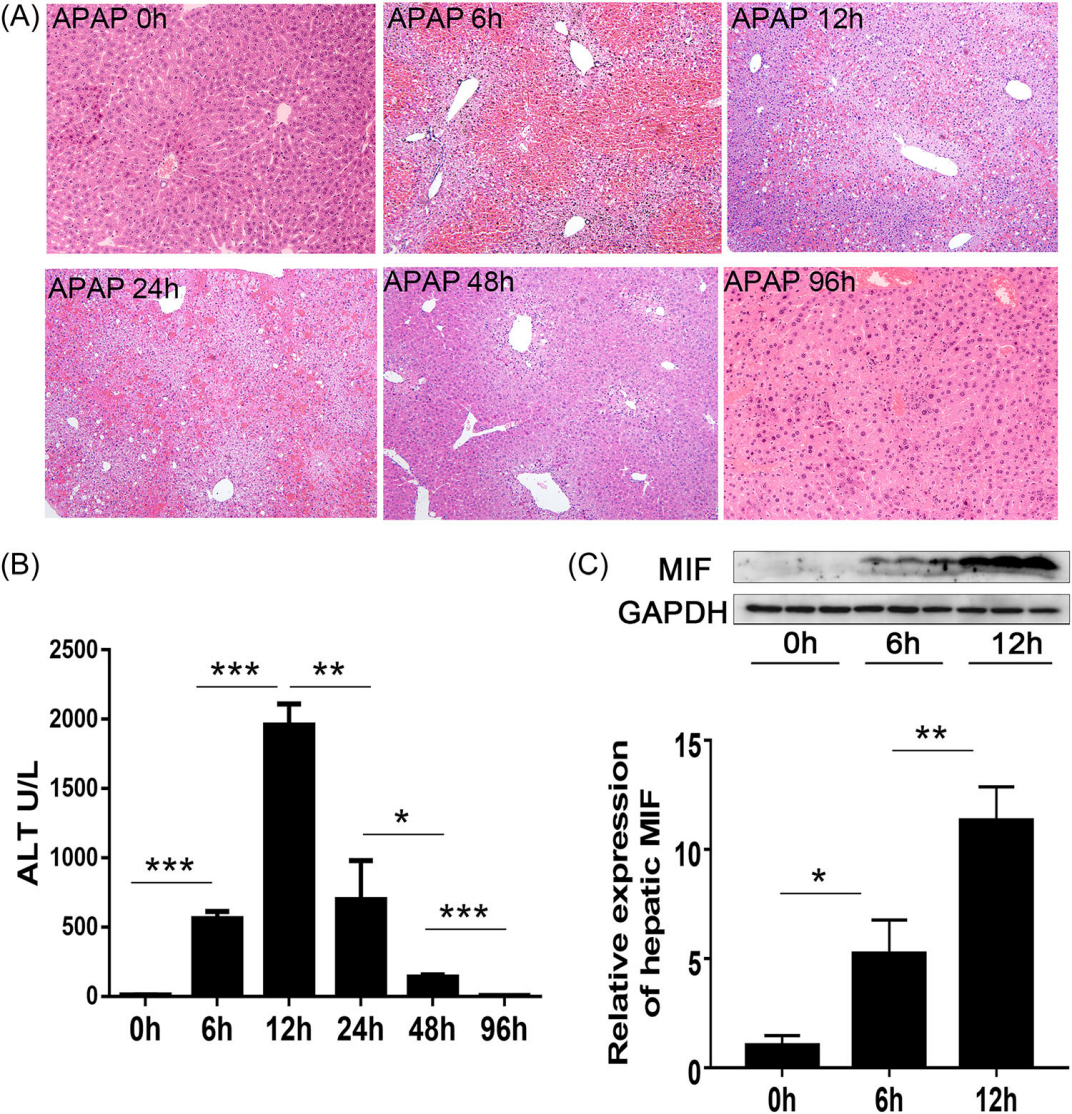
Acetaminophen (APAP)‐induced acute liver failure (ALF) in mice. (A) Liver histopathological findings (liver tissue) indicate ALF after APAP injections (hematoxylin and eosin staining, ×100). (B) Serum alanine aminotransferase (ALT) levels after APAP injection. (C) Migration inhibitory factor (MIF) expression in the liver over time. *p < .05, **p < .01, ***p < .001. GAPDH, glyceraldehyde 3‐phosphate dehydrogenase (control).

Compared with the control group, massive hepatocyte necrosis and lymphocyte infiltration occurred shortly after APAP challenge in the liver of APAP‐induced ALI mice (Figure 1A,B). The ALT level also sharply increased, and approximately 50% of the mice died within 12 h post‐APAP challenge. However, the surviving mice gradually recovered from the inflammation after the acute phase. As a result, the mouse model replicates the pathophysiology of clinical ALF.

Moreover, MIF expression in the liver of the APAP‐induced ALI mice increased as liver inflammation increased over the same 12‐h period, then decreased during the recovery period (Figure 1C). These results suggest that MIF may play an essential role in promoting the progression of liver inflammation in APAP‐induced ALI.

### 3.2 HDCs experiences apoptosis along with the progression of APAP‐induced liver injury

**Figure 2:**
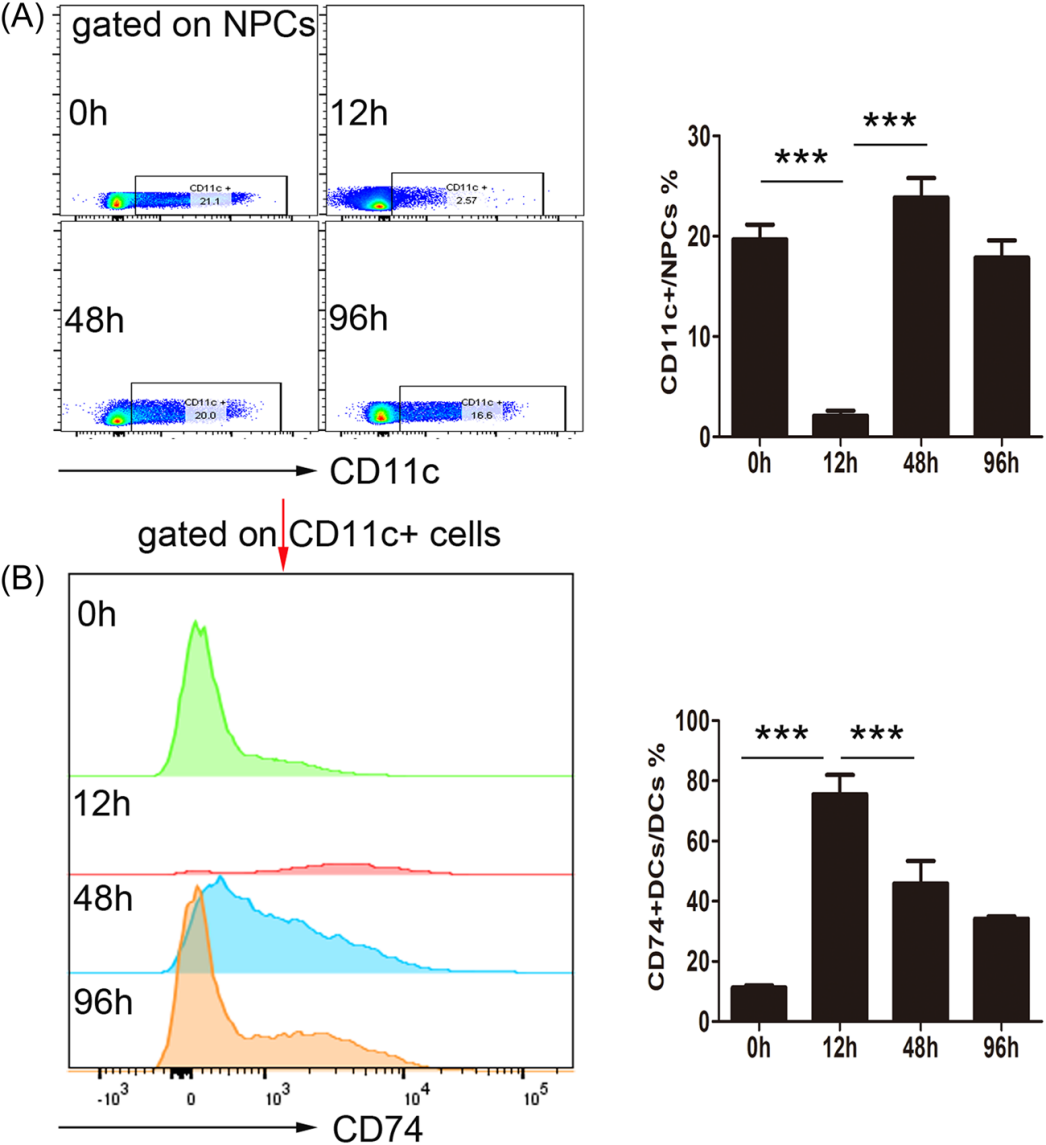
Hepatic dendritic cells (HDC) and cluster of differentiation (CD) 74 expression changes over time in mice with acute liver failure. (A) The proportion of HDCs in CD45 + cells; the left panel presents a representative flow cytometry diagram. (B) CD74 expression on HDCs. The left panel presents a representative diagram of flow cytometry fluorescence intensity. *p < .05, **p < .01, ***p < .001. DC, dendritic cell; NPC, nonparenchymal cells.

After injecting APAP, there was a sharp reduction in the number of HDCs during the peak period of inflammation (Figure 2A), but CD74 expression (a MIF ligand) on HDCs significantly increased (Figure 2B). However, CD74 expression decreased during the recovery period, whereas the number of HDCs gradually increased. This result suggests that HDCs are depleted during APAP‐induced ALI, which may indicate that they have a protective role against inflammation. Conversely, high CD74 expression may promote HDC depletion at the peak of inflammation, further progressing the disease.

### 3.3 Adoptive transfer of BMDCs has a protective effect against APAP‐induced liver injury

**Figure 3:**
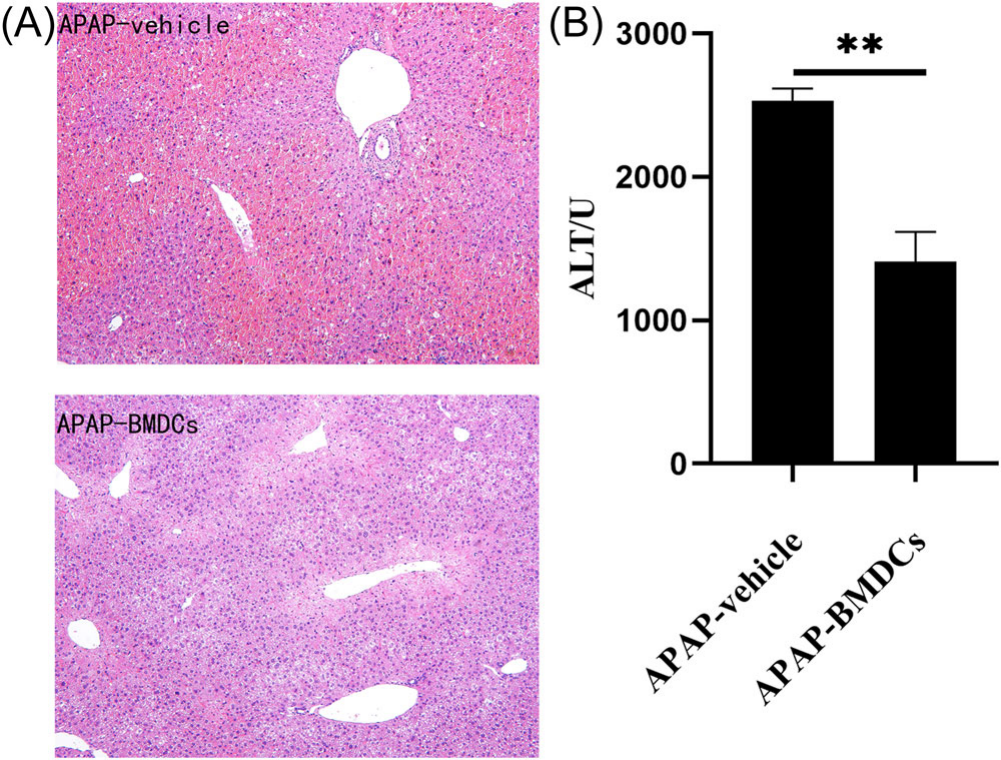
Supplementing mice with acetaminophen (APAP)‐induced acute liver failure with bone marrow‐derived dendritic cells (BMDCs) has a protective effect. (A) The histopathological findings (liver tissue) from mice who did and did not receive BMDCs (hematoxylin and eosin staining, ×100) after 12 h. (B) Serum alanine aminotransferase (ALT) levels in who did and did not receive BMDCs after 12 h. *p < .05, **p < .01, ***p < .001. PBS, phosphate‐buffered saline (i.e., control).

We transferred adoptive BMDCs into the tail vein of the mice to verify the role of HDCs in APAP‐induced ALI development. Mice in the adoptive transfer group had significantly less liver damage than those in the control group (Figure 3A,B), suggesting that HDCs protect mice from APAP‐induced ALI.

**Figure 4:**
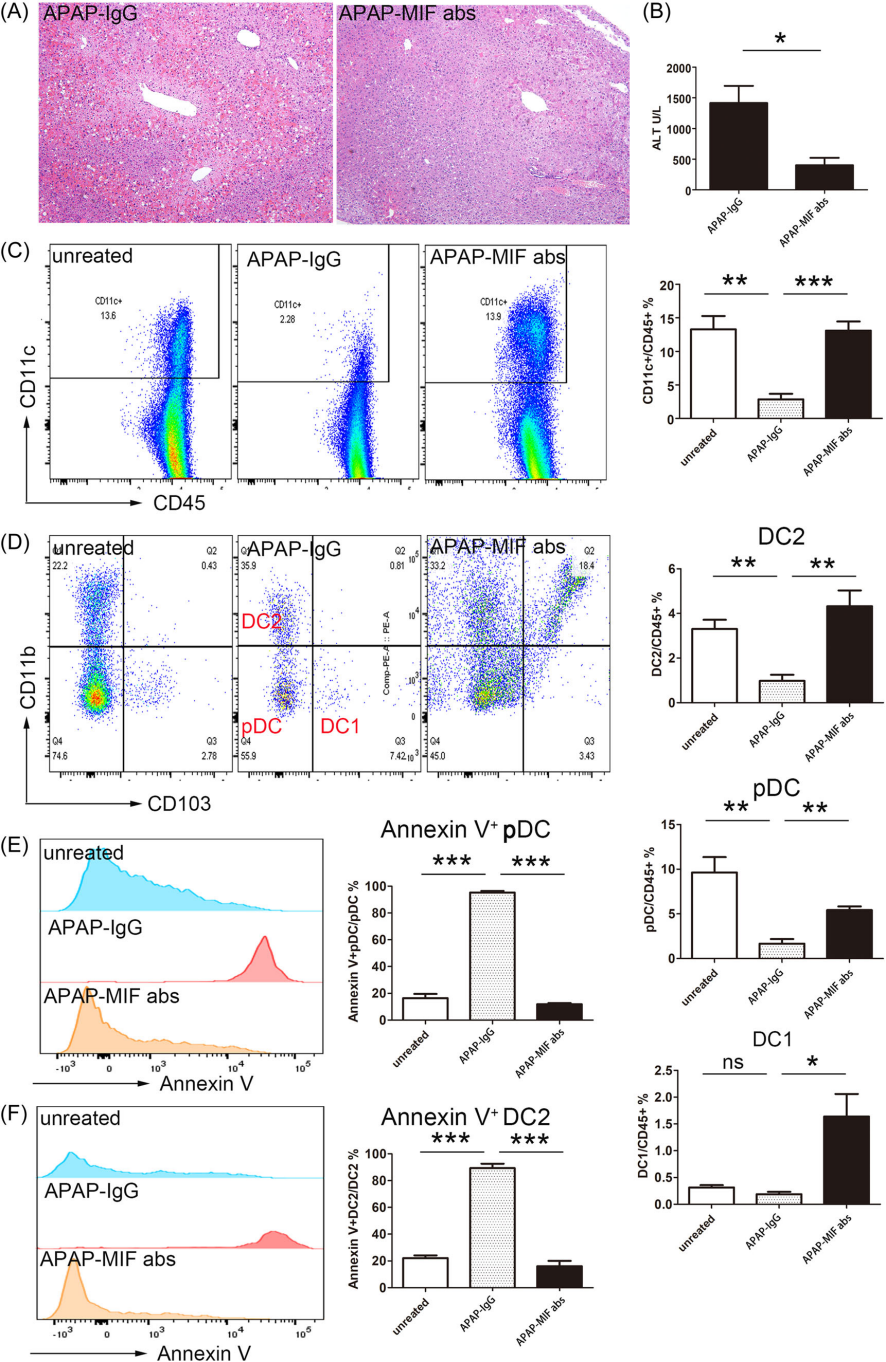
Mice with acetaminophen (APAP)‐induced acute liver failure have less liver damage after supplementing with migration inhibitory factor (MIF) antibodies. (A) Histopathological findings (liver tissue) 12 h after MIF antibody injections (hematoxylin and eosin staining, ×100). (B) Serum alanine aminotransferase (ALT) levels 12 h after MIF antibody treatment. (C) The proportion of hepatic dendritic cells (HDCs) and (D) HDC subgroups (plasmacytoid [pDC] and DC2) 12 h after treatment. (E) Annexin V expression changes in pDC. (F) DC2 cells 12 h after treatment. The left panels in (E) and (F) are representative flow cytometry images. DC, dendritic cells; IgG, immunoglobin G.

The MIF antibody also had a significant protective effect during APAP‐induced ALI (Figure 4A,B), evidenced by a significant increase in the number of HDCs in the MIF antibody group compared with the control group. Furthermore, the HDC apoptosis rate significantly decreased and the numbers of pDC and DC2 cells (HDC subgroups) in the liver significantly increased in the antibody injection group compared with the control group (Figure 4C,D). Furthermore, the liver's pDC and DC2 apoptosis rates were significantly higher in the ALF mice than in the control mice. Also, the apoptosis rate significantly decreased in the ALF mice after MIF antibody injection compared with ALI mice without MIF antibodies (Figure 4E,F). This result implies that the high hepatic MIF expression promotes the disease progression during ALF development. Likely, this is due to the depletion of DCs (e.g., pDC and DC2) through the MIF/CD74 pathway.

## 4 DISCUSSION

Hepatotoxic drugs are common in clinical practice, as are hepatotoxic herbs and dietary supplements, and drug‐induced ALF can cause deterioration and more severe complications, complicating treatment. Furthermore, sensitive early warning indicators and specific interventions are lacking, perpetuating the high mortality rate.

It is widely thought that, apart from direct drug and metabolite‐induced cellular stress, abnormal immunizations are a primary cause of DILI in certain situations.^2^ Pathomorphological and immunological studies have indicated that early microcirculation disorders and cell apoptosis are critical to the progression of ALF and liver cell necrosis in a short period and are closely related to host immune damage. However, the specific mechanisms remain unknown.^16^, ^17^

The immunomodulatory effect of MIF after a liver injury has been reported in many liver diseases. The latest research suggests that treating mice with the MIF antagonist, ISO‐1, improved liver recovery after APAP‐induced injury (i.e., nonliver failure) by hindering the production of receptor‐interacting protein kinase 3 (i.e., RIPK3) and heat shock protein 70 after oxidative stress and neutrophil recruitment.^18^ Studies have demonstrated that MIF promotes liver inflammation damage by mediating T cell activation in a concanavalin‐A (ConA)‐mediated immune liver injury model.^10^ Our study administered excessive APAP to induce ALF in mice, finding that MIF expression in the liver rapidly increased with the exacerbation of liver inflammation. However, there was significantly less inflammation in the mice injected with MIF‐specific antibodies in the early stage of inflammation than those without MIF antibodies, confirming that MIF promotes APAP‐induced ALI progression. This finding is consistent with the previous study showing that APAP hepatotoxicity was attenuated in MIF‐/‐ mice.^19^ Our study indicated that MIF‐specific interventions may optimize ALF treatment strategies.

The role of DCs in APAP‐induced ALI progression has also become more evident in recent years. Mouse studies have confirmed that HDCs have a protective role regarding APAP‐induced ALI. We found that liver inflammation and the mortality rate increased in mice with APAP‐induced ALI after depleting HDCs. Conversely, the endogenous expansion of HDCs protected mice from APAP‐induced liver damage. Furthermore, mechanistic studies reported that HDCs inhibit NK cell activation and induce neutrophil apoptosis.^20^ Similarly, in a Con‐A‐induced immune ALI model, liver inflammation worsened after selective depletion of pDCs but improved after transferring and adopting pDCs. Mechanistic studies suggest that pDCs have a protective function primarily due to IL‐35 secretion.^8^ We found that the number of HDCs decreased sharply during the peak period of inflammation in mice with ALF, impeding the protective effect of HDCs. However, liver inflammation improved after supplementing with BMDCs. In our study, we discovered that liver DC itself experienced apoptosis during the progression of liver injury, and adoptive transfer of BMDCs significantly alleviated hepatic damage, suggesting that hepatic DCs apoptosis exacerbates APAP hepatotoxicity. This finding is consistent with the Connolly's conclusion,^20^ but it also adds value due to its alternative point of view.

This study also found that CD74 (a MIF ligand) was highly expressed on HDCs during the phase of HDC depletion and apoptosis, which is an interesting phenomenon. This result verified the MIF's proinflammatory effect, suggesting that MIF may activate HDC apoptosis through CD74. Consequently, if HDCs are depleted, their protective effect is hindered, and disease progression occurs. After injecting the ALF mice with MIF antibodies, the numbers of HDCs in the liver considerably increased, especially pDC and DC2 cells, further confirming the apoptotic function of MIF. Several studies have reported the role of MIF‐mediated apoptosis. For example, Zheng et al. demonstrated that fenofibrate attenuates insulin β‐cell dysfunction induced by fatty acids and apoptosis by inhibiting the nuclear factor kappa B/MIF pathway.^21^ Our study did not explore the MIF/CD74‐mediated mechanisms of HDC apoptosis at the cellular level, which is a topic for future studies.

This study found that MIF bound to CD74 expressed on HDCs mediates HDC apoptosis, which accelerates APAP‐induced ALI, leading to ALF in mice. However, blocking the MIF/CD74 pathway had a protective effect in mice with ALF. Therefore, this study provides evidence related to the immune damage mechanisms of ALF, which might benefit treatment development.

## AUTHOR CONTRIBUTIONS

**Wu Zezhou**: Investigation (equal); data curation (equal); formal analysis (equal); writing—original draft (lead); methodology (equal); writing—review and editing (equal). **Luan He**: Data curation (equal); resource (equal); investigation (equal); writing—review and editing (equal). **Huang Jinghui**: Software (lead); investigation (equal); writing—review and editing (equal). **Liao Boming**: Conceptualization (equal); methodology (equal); formal analysis (equal); project administration (equal); writing—review and editing (equal); supervision (equal). **Xiao Fang**: Conceptualization (equal); methodology (equal); formal analysis (equal); project administration(equal); writing—review and editing (equal); supervision (equal); funding acquisition (lead).

## CONFLICT OF INTEREST STATEMENT

The authors declare no conflict of interest.

## ETHICS STATEMENT

The Animal Experiment Ethics Committee of the Animal Experiment Center of Guangxi Medical University approved this study (No. 202112001). The production and use license numbers are SCXK (GUI) 2020‐0003 and SYXK (GUI) 2020‐0004, respectively.

## Data Availability

The data that support the findings of this study are available from the corresponding author upon reasonable request.
